# Increased mortality in socioeconomic disadvantaged municipalities during the first phase of the COVID-19 pandemic in Lombardy region

**DOI:** 10.1093/eurpub/ckae054

**Published:** 2024-03-28

**Authors:** Gianfranco Alicandro, Alberto Gerli, Carlo La Vecchia

**Affiliations:** Department of Pathophysiology and Transplantation, Università degli Studi di Milano, Milan, Italy; Department of Paediatrics, Cystic Fibrosis Centre, Fondazione IRCCS Ca’ Granda Ospedale Maggiore Policlinico, Milan, Italy; Department of Clinical Sciences and Community Health, Università degli Studi di Milano, Milan, Italy; Department of Clinical Sciences and Community Health, Università degli Studi di Milano, Milan, Italy

## Abstract

**Background:**

Lombardy was the first European region most severely affected by the coronavirus disease 2019 pandemic in the spring of 2020. During that period, a substantial increase in socioeconomic inequality in total mortality was observed. This study aims to evaluate mortality data in the region up to September 2023 to verify whether the increased disparities between the poorest and the wealthiest municipalities persisted in the subsequent phases of the pandemic.

**Methods:**

This study analyzed mortality data from January 2019 to September 2023 in Lombardy's municipalities by month and pandemic phases characterized by the predominance of the different severe acute respiratory syndrome coronavirus 2 (SARS-CoV-2) variants. Municipalities were grouped according to the average income or pension of their residents. Age-standardized mortality rates (ASMRs) and the ASMR ratio between the poorest and the wealthiest municipalities were compared throughout the study period.

**Results:**

In the pre-pandemic period (January 2019 - February 2020), the ASMR ratio at all ages between the poorest and the wealthiest municipalities fluctuated between 1.12 [95% confidence interval (CI): 1.07–1.16] and 1.29 (95% CI: 1.25–1.34). In March 2020, the ASMR ratio increased to 1.49 (95% CI: 1.45–1.52 95%) and returned to values registered before the pandemic thereafter. A similar pattern was observed in the analysis of mortality ≥ 65, using the average pension for group municipalities.

**Conclusions:**

During the dramatic circumstances that the region faced in March 2020, pre-existing socioeconomic inequalities substantially widened. With the reorganization of the health system and the availability of vaccines, these disparities returned to the levels recorded before the pandemic.

## Introduction

The coronavirus disease 2019 (COVID-19) pandemic caused approximately 100 000 excess deaths in Italy in 2020, 60 000 in 2021 and 66 000 in 2022, while no excess was registered in the first semester of 2023.^1^ However, important geographic heterogeneity was observed in the country, particularly in the first phase of the pandemic (March–April 2020), when the majority of the excess deaths (25 000 out of 45 000 in the entire country) were registered in the Lombardy region.^2^

Lombardy is a highly populated and wealthy region located in the North of Italy, where approximately 10 million inhabitants (one-sixth of the Italian population) live. In March 2020, the region had to face a substantial surge in COVID-19 cases, leading to a massive demand for hospitalization and intensive care unit beds.^3^

Area-level indicators of socioeconomic deprivation have been found to be associated with the risk of COVID-19-related hospitalization and death, adding additional risk to individual risk factors.^4^^,^^5^

In a previous work,^6^ we documented an important increase in income inequalities in total mortality in the first phase of the pandemic in Lombardy. The mortality ratio between the poorest and wealthier municipalities ranged between 1.12 and 1.33 before the pandemic (from January 2019 to February 2020), but increased to 1.61 in March 2020.

In the current work, we collected new data up to September 2023, to verify whether the excess mortality in disadvantaged areas, observed in the first phase of the COVID-19 pandemic, persisted in the subsequent phases.

## Methods

Death counts, disaggregated by sex, age and month, that occurred in the municipality of the Lombardy region were collected from a period spanning from January 2019 to September 2023. The data were obtained from the provisional daily mortality statistics published by the National Institute of Statistics (Istat).^7^ Additionally, population counts, disaggregated by sex, age group and municipality of the Lombardy region, were retrieved from the Istat archives.^8^

Average municipal taxable income and retirement incomes for each year from 2019 to 2022 were obtained from the Tax Register of the Ministry of Economy and Finance.^9^ These data were available for 1506 out of 1507 municipalities throughout the study period, except for the municipality of Vendrogno.

Municipalities were grouped based on the quintiles of average income or pension distributions for the year preceding the mortality data. For each group, age-specific mortality rates were calculated monthly using the number of registered deaths and person-days at risk. Age-standardized mortality rates (ASMRs) and their standard errors were computed monthly using the direct method, applying the World Health Organization standard population distribution to the age-specific mortality rates computed for each calendar year. ASMRs were computed for all age categories and for ages ≥65 years.^10^

Differences in the ASMR and ratios between the ASMRs of the first and the last group of municipalities, classified by income and pension quintiles, were calculated monthly and for different phases of the COVID-19 pandemic. The 95% confidence intervalsfor the ASMR ratio were computed using the method reported in Boyle and Parkin.^10^

Pandemic phases were defined as follows: phase 1 (March–April 2020), phase 2 (Oct 2020–May 2021), phase 3 (July 2021–December 2021, characterized by the predominance of the Delta variant) and phase 4 (January 2022–December 2022, characterized by different lineages of the Omicron variant). The years 2019 and 2023 were evaluated as separate periods for comparative analysis.

As a sensitivity analysis, we restricted the analysis to municipalities with a population size of <10 000 inhabitants.

## Results

From January 2019 to September 2023, 540 909 deaths were registered in the Lombardy region at all ages and 460 590 at ages ≥65 years (85.2% of all deaths). The ASMRs increased remarkably from 2019 to phase 1 of the pandemic in all municipality groups. This increase persisted up to phase 3 of the pandemic. While during phase 4 it returned to the pre-pandemic values. A similar trend was observed when we analyzed mortality at ages ≥65 years (table 1).

**Table 1:** ASMRs per 100 000 person-days (number of deaths) at all ages and ages ≥65 years in the municipalities of the Lombardy region grouped according to the average income of pension of their residents in 2019 and during the different phases of the COVID-19 pandemic

		Groups of municipalities classified according to quintiles of average income or pension				
SES indicator	Period	1	2	3	4	5
Income	2019	3.30 (5321)	2.99 (8991)	2.59 (16 387)	2.54 (19 010)	2.77 (50 098)
	Phase 1	8.30 (2202)	9.28 (5135)	7.70 (7948)	6.81 (8324)	6.18 (18 815)
	Phase 2 (Alpha)	3.91 (4230)	3.39 (7510)	3.25 (13 691)	3.11 (15 199)	3.45 (42 138)
	Phase 3 (Delta)	3.24 (8062)	3.02 (15 881)	2.73 (25 973)	2.71 (30 317)	2.95 (80 697)
	Phase 4 (Omicron)	3.36 (5545)	3.16 (11 059)	2.98 (17 519)	2.84 (21 089)	3.10 (55 204)
	2023	2.94 (3625)	2.90 (7587)	2.66 (11 686)	2.53 (14 072)	2.76 (36 788)
Pension	2019	14.99 (4656)	14.42 (7985)	13.68 (11 586)	13.54 (14 066)	13.30 (46 093)
	Phase 1	44.00 (2336)	47.96 (4445)	40.84 (6152)	35.11 (6034)	30.72 (18 104)
	Phase 2 (Alpha)	16.93 (3696)	16.90 (6451)	16.26 (9863)	16.41 (11 495)	16.53 (39 120)
	Phase 3 (Delta)	14.97 (7371)	14.54 (13 077)	14.15 (19 104)	14.17 (22 861)	14.13 (74 926)
	Phase 4 (Omicron)	15.87 (5080)	14.98 (8959)	14.73 (13 126)	14.74 (15 808)	14.85 (51 547)
	2023	13.43 (3251)	13.41 (6062)	13.09 (8819)	12.93 (10 507)	13.02 (33 983)

SES, socioeconomic status.

In 2019, the ASMR ratio between the poorest and richest municipalities was 1.19 (95% CI: 1.18–1.20) at all ages and 1.13 (95% CI: 1.10–1.16) at ages ≥65 years. During the first phase of the pandemic they increased to 1.34 (95% CI: 1.32–1.35) at all ages and to 1.43 (95% CI: 1.37–1.49) at ages ≥65 years. During the subsequent phases of the pandemic, the ASMR ratio returned to values slightly lower than those registered in 2019.

**Figure 1:**
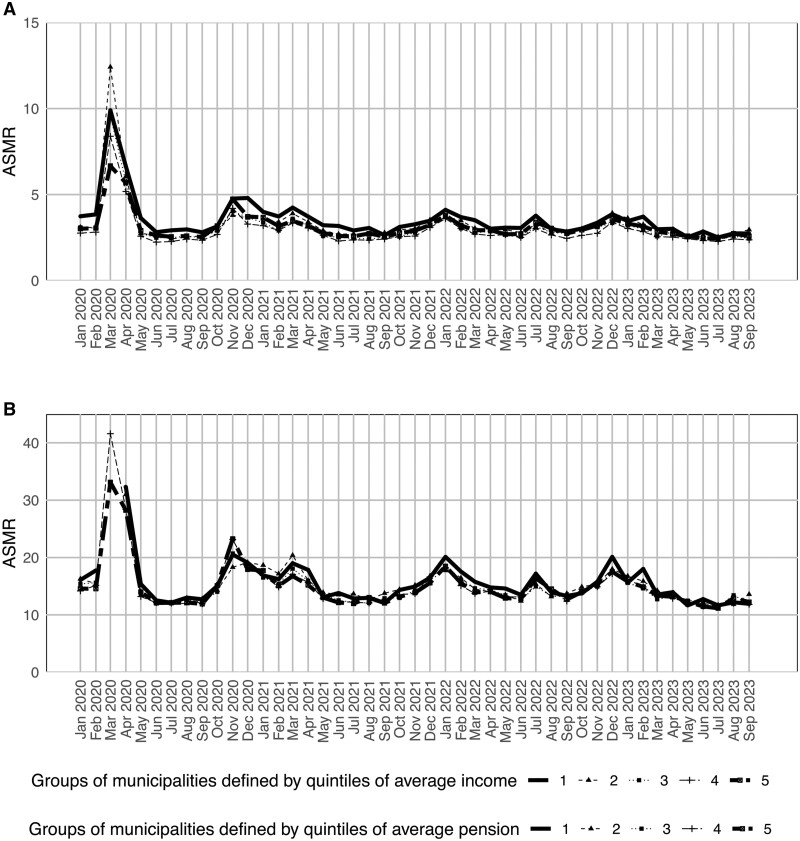
Monthly age-standardized mortality rate (per 100 000 person-days) at all ages and at ages ≥65 years in the municipalities of the Lombardy region grouped according to quintiles of average income or pension of their residents. (A) ASMR by income, all ages. (B) ASMR by pension, all ages.

Figure 1 shows the trend in the ASMRs during the study period. The ASMR peaked in March 2020 for all municipality groups, with, as expected, a seasonality pattern characterized by higher mortality in the winter months and in July 2022, when the region faced an important heat wave.

**Figure 2:**
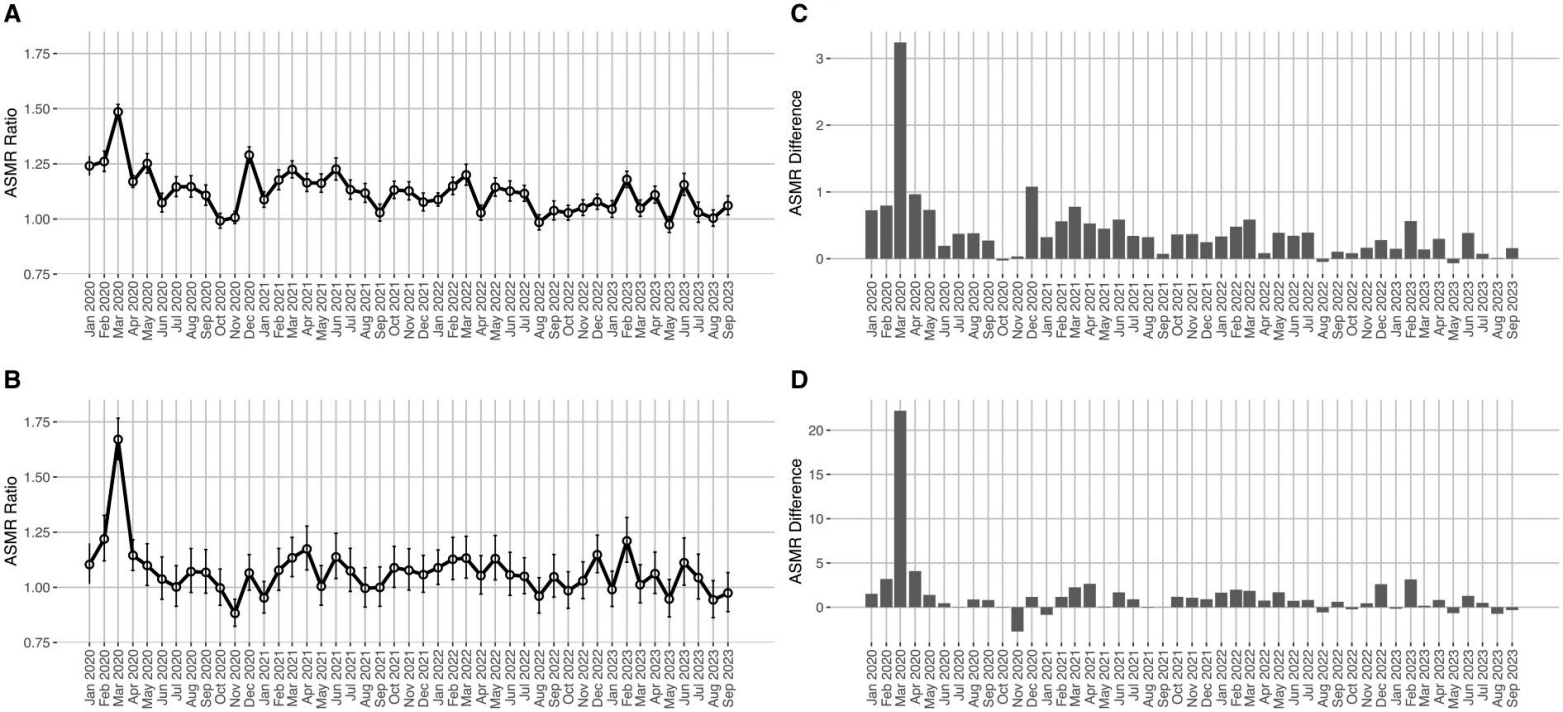
Ratio and difference in the age-standardized mortality rate (per 100 000 person-days) at all ages and at ages ≥65 years between the poorest and the wealthiest municipalities of the Lombardy region grouped according to the average income or pension of their residents. (A) ASMR ratio by income, all ages. (B) ASMR ratio by pension, age ≥65 years. (C) ASMR difference by income, all ages (D): ASMR difference by pension, age ≥65 years.

Figure 2 gives the ratio and the difference in the ASMR between the poorest and the richest municipalities. In the income-based analysis, the ASMR ratio for all ages fluctuated between 1.12 (95% CI: 1.07–1.16) and 1.29 (95% CI: 1.25–1.34) during the pre-pandemic period (January 2019–February 2020). It increased to 1.49 in March 2020 and subsequently returned to values comparable to the pre-pandemic period. A similar pattern emerged in the pension-based analysis, with the ASMR for ages ≥65 years ranging between 0.98 (95% CI: 0.89–1.07) and 1.25 (95% CI: 1.15–1.37) before the pandemic. It peaked in March 2020, reaching 1.67 (95% CI: 1.58–1.77) for ages ≥65 years, and declined thereafter. In absolute terms, the differences between the ASMR at all ages for the poorest and highest municipalities peaked in March 2020 exceeding 3 deaths per 100 000 person-days, but remained below 1 death per 100 000 person-days thereafter. Similarly, the ASMR at ages above 65, peaked in March 2020, exceeding 20 deaths per 100 000 person-days, and in the subsequent months, it stayed below 5 deaths per 100 000 person-days.

**Figure 3:**
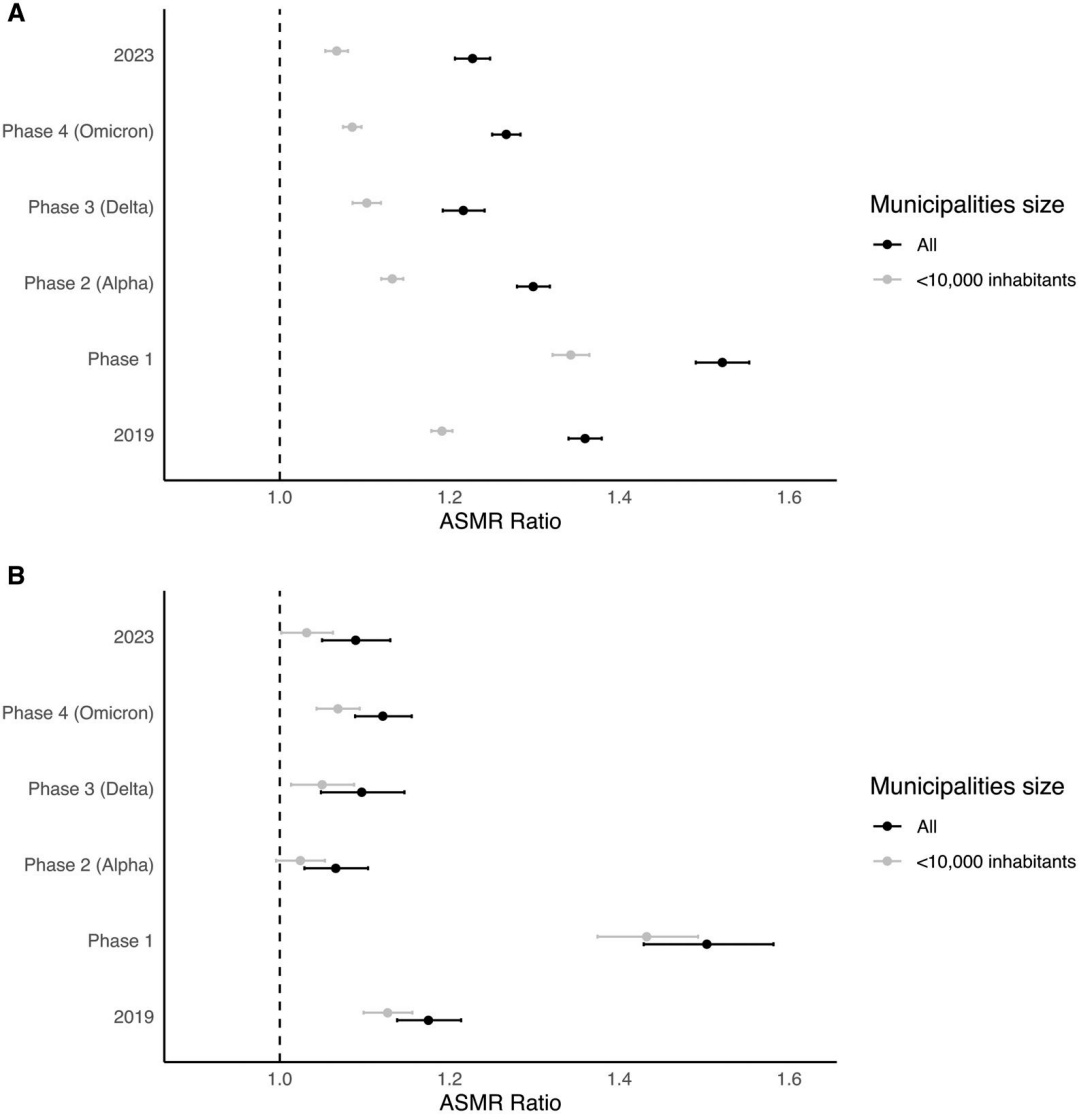
Comparison of the age-standardized death rate ratio between the poorest and wealthiest municipalities of the Lombardy region obtained by including all the municipalities or restricting the analysis to municipalities with population size <10 000 inhabitants. (A) ASMR by income, all ages. (B) ASMR by pension, all ages.

Figure 3 shows the results of the sensitivity analysis, comparing the estimates of the ASMR ratio between the richest and the poorest municipalities, obtained including all municipalities of the region or only 1315 population size of <10 000 inhabitants. The 1316 municipalities with <10 000 inhabitants represent 87.3% of all municipalities in the region, housing a total population of 4 010 344 inhabitants. In the case of average income and all-age mortality, the ASMR ratio estimate was lower for municipalities with a population <10 000 as compared with the whole of Lombardy. Conversely, no significant disparities were noted in the analysis for ages ≥65 years when using average pensions to categorize municipalities.

## Discussion

We documented a significant widening of socioeconomic inequality in the municipalities of the Lombardy region during March 2020. This month was marked by a sudden surge in infections, leading to a tremendous crisis in hospitals that were ill-prepared for such an event.

The first case in the region was identified on 20 February 2020, involving a 38-year-old previously healthy man with no known underlying health conditions admitted to the Hospital of Codogno in the province of Lodi. He presented with mild pneumonia, resistant to therapy. By February 2023, recreational and teaching activities were suspended in the municipalities with at least one positive case. In the subsequent days, public health authorities imposed further restrictions, including the cancellation of all public events across the entire region on February 25. Over the following weeks, the Codogno area, along with several neighbouring towns in southern Lombardy, witnessed a rapid increase in the number of detected cases. Responding to this surge, local, regional and national health authorities implemented intensive testing, contact tracing, isolation of confirmed cases, and quarantine of case contacts to prevent the spread of the infection. In those municipalities, public manifestations, non-essential commercial, recreational, sports, work and teaching activities, as well as public transport, were suspended. On March 1, access to the 10 municipalities identified as the epicentre of the ongoing outbreak was forbidden. Subsequently, a regional lockdown was enforced on March 8, followed by a national lockdown on 11 March 2020.

Our results align with those reported in a study conducted in Emilia-Romagna,^11^ another region located in the North of the country that, following Lombardy, was severely hit by the pandemic. The study found that people living in the most disadvantaged census blocks experienced an increased risk of overall mortality (+23% among men and +12% among women) and COVID-19 death (+39% among men and +55% among women) during the outbreak of the pandemic in March–April 2020.

The widening of socioeconomic inequalities documented in our and other studies can be attributed to different factors related to individual behaviours and the heterogeneous impact of the pandemic per se, or the indirect effect on the health systems.

A French study^12^ in March–April 2020 reported a 30% increase in the excess mortality in poorest municipalities, defined by a median income below the first quintile, compared with wealthier municipalities. This excess mortality was concentrated in April 2020, particularly in the severely impacted areas of the Northeast of the country. To a lesser extent, this difference persisted during the second wave of the pandemic (October–December 2020).

The similarity between these findings and our results is particularly noteworthy, given the shared patterns of uneven epidemic spread within Italy and France and similarities in the strength and timing of the implementation of containment measures. In France, the first wave mainly affected the Northeast, where 80% of excess mortality occurred, despite only 44% of the total urban population living in the region. Conversely, the West and the South were almost spared. Similarly to Italy, a national lockdown was enforced on March 17 and lasted until May 11, with all non-essential workers staying home.

Disadvantaged individuals may have been at higher risk of infection due to lower awareness of the potential harm of COVID-19 or an increased frequency of contacts during their work activities.^13–15^ They might have also underestimated the severity of COVID-19 symptoms, potentially leading to delays in seeking medical aid. Moreover, the higher prevalence of comorbidities in individuals living in poor municipalities may have exposed them to a higher risk of severe COVID-19 and to the adverse consequences of overstressed health systems.^16^

Interestingly, the French study^12^ found that the restriction of contacts induced by the lockdowns did not appear to have had a major role in sustaining income inequality during the pandemic. Instead, the share of essential workers and overcrowded houses mediated most of the differences in excess mortality between the poorest and wealthier municipalities.

After the peak in the ASMR observed in March 2020, during the subsequent waves, the disparities between the poorest and the wealthiest municipalities in the Lombardy region returned to the levels registered before the pandemic. By April 2020, the ASMR ratio levelled off, despite a substantial excess mortality observed in that month.^2^ This phenomenon was likely due to the reorganization of the healthcare system in the months following the health crisis of March 2020 and the distribution of vaccines in the spring of 2021, with priority given to clinically vulnerable and older populations.

The ecological nature of the study should be considered when interpreting the results.^17^ Using the average income in areas with thousands of inhabitants may not capture the granularity of socioeconomic deprivation within the municipalities. On the other hand, municipalities are the smallest administrative entities in our countries. They represent crucial units for identifying areas that require close monitoring and timely intervention during extraordinary events, with significant implications for public health.

In conclusion, during the dramatic period that the region faced in March 2020, pre-existing socioeconomic inequalities substantially widened. However, by April 2020, they had already returned to the levels registered before the pandemic. This finding underscores the importance of considering area-level socioeconomic indicators in policies aimed at addressing unforeseen events, such as the COVID-19 pandemic. This is crucial for mitigating the disproportionate effects that such events have on the most disadvantaged individuals.

## Data Availability

The data used in this study were derived from sources in the public domain. Key points In March 2020, Lombardy was among the European regions most severely affected by the COVID-19 pandemic. During that month, pre-existing socioeconomic inequalities in mortality significantly increased in the poorest municipalities of the region. In the subsequent months, socioeconomic inequalities in mortality returned to pre-pandemic levels.
